# Supplementary material for: Development and validation of a risk prediction tool for drug-related problems in pre-operative elective surgical patients (mediPORT): A case-control study
Source: PLoS One. 2025 Sep 2;20(9):e0326088. doi: 10.1371/journal.pone.0326088 (PMC12404507; doi:10.1371/journal.pone.0326088)
Supplement: S1 Appendix — (DOCX) [file pone.0326088.s002.docx]

**S1 Appendix:** Full details of tested predictors for the model development

*Patients’ demographic characteristics*

1. Age in years
2. Sex assigned at birth
3. Main residence
   urban/rural typology was assessed through postal codes via classification systems of "*Urban-Rural Typology, Statistics Austria*" and the "*German Federal Institute for Research on Building, Urban Affairs and Spatial Development*".
   For patients who did not come from Austria or Germany, no urban/rural typological assignment was performed (missing data).

*Physical examination of patients by anaesthetists*

1. Body Mass Index (BMI) in kg/m^2^
2. Intolerance

For patients with intolerance, a distinction was made between drugs, other intolerances and no intolerances.

1. Allergy

For patients with allergy, a distinction was made between allergy and no allergy.

Additionally, the allergen/intolerance trigger was documented (e.g. penicillin).

1. Pre-operative risk via the American Society of Anesthesiologists (ASA) classification
   The ASA classification system is used to predict the perioperative risks of patients from ASA 1 (healthy patient) to ASA 6 (death). Patients with ASA 5 and 6 were excluded from the study [2].

*Disease characteristics*

1. Comorbidity assessed via the Charlson Co-morbidity Index (CCI)

The CCI represents a valid assessment tool to predict long-term mortality of patients [3]. The index contains 17 categories of comorbidities based on the International Statistical Classification of Diseases and Related Health Problems (ICD)-10 diagnoses. Categories are weighted from 1 to 6. For the CCI assessments, history of hospital admissions of patients within the last five years prior to the PAC admission date was searched.

1. Hospital speciality clinic for elective surgery

*Previous treatments*

1. Hospitalization (ambulatory/inpatient treatment) within the previous 12 months

*Routine laboratory test results*

1. Renal function, estimated glomerular filtration rate (eGFR)

eGFR was categorized according to the KDIGO (Kidney Disease: Improving Global Outcomes) 2012 clinical practice guideline for the evaluation and management of chronic kidney disease through Chronic Kidney Disease (CKD) stages 1 (kidney function normal or high) to 5 (kidney failure) [4].

*Medical history*

1. Number of drugs at admission

The number included long-term medication, short-term medication as well as on-demand medication and over-the-counter medication.

**References:**

1. PCNE. Pharmaceutical Care Network Europe, Position Paper on the PCNE definition of Medication Review 2016. <https://www.pcne.org/upload/files/149_Position_Paper_on_PCNE_Medication_Review_final.pdf> (accessed 06.06.2023).

2. ASA Physical Status Classification System, 2020. <https://www.asahq.org/standards-and-practice-parameters/statement-on-asa-physical-status-classification-system> (accessed 1.02.2021).

3. Charlson ME, Pompei P, Ales KL, MacKenzie CR. A new method of classifying prognostic comorbidity in longitudinal studies: development and validation. *J Chronic Dis* 1987; **40:** 373-83.

4. KDIGO. Clinical practice guideline for the evaluation and management of chronic kidney disease. *Kidney Int Suppl.* 2012; **3:1-150.**
