# Supplementary material for: Development and validation of a risk prediction tool for drug-related problems in pre-operative elective surgical patients (mediPORT): A case-control study
Source: PLoS One. 2025 Sep 2;20(9):e0326088. doi: 10.1371/journal.pone.0326088 (PMC12404507; doi:10.1371/journal.pone.0326088)
Supplement: S2 Appendix — (DOCX) [file pone.0326088.s003.docx]

**S2 Appendix: Boxplots and barplots of participant characteristics**

[**Figure 1:** Distribution of age (in years) among case- and control-patients. 1](#_Toc155273324)

[**Figure 2:** Sex (defined as sex at birth) among case- and control patients 2](#_Toc155273325)

[**Figure 3:** Residence among case- and control patients. 3](#_Toc155273326)

[**Figure 4:** Allergy among case- and control patients. 4](#_Toc155273327)

[**Figure 5:** Intolerance among case- and control patients.. 5](#_Toc155273328)

[**Figure 6:** Number of drugs at admission among case- and control patients. 6](#_Toc155273329)

[**Figure 7:** American Society of Anesthesiologists (ASA) classification among case- and control patients. 7](#_Toc155273330)

[**Figure 8:** Body Mass Index (BMI) among case- and control patients. 8](#_Toc155273331)

[**Figure 9:** Hospitalisation in the last 12 months among case- and control patients. 9](#_Toc155273332)

[**Figure 10:** Charlson Comorbidity Index (CCI) among case- and control patients 10](#_Toc155273333)

[**Figure 11:** Hospital ward among case- and control patients. 11](#_Toc155273334)

[**Figure 12:** Renal function groups among case- and control patients 12](#_Toc155273335)

[**References:** 13](#_Toc155273336)

### **Figure 1: Distribution of age (in years) among case- and control-patients.**


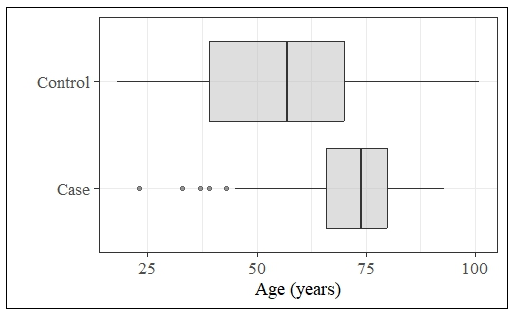


### **Figure 2: Sex (defined as sex at birth) among case- and control patients. F= female, M= male.**


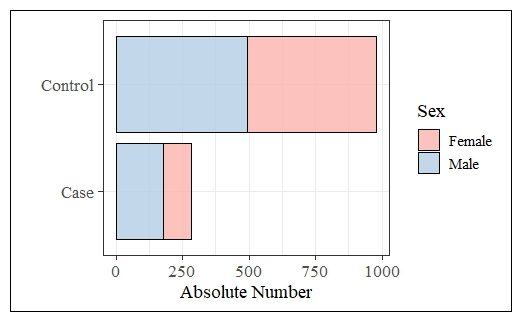


### **Figure 3: Residence among case- and control patients. R=rural, U=urban.** [1, 2]


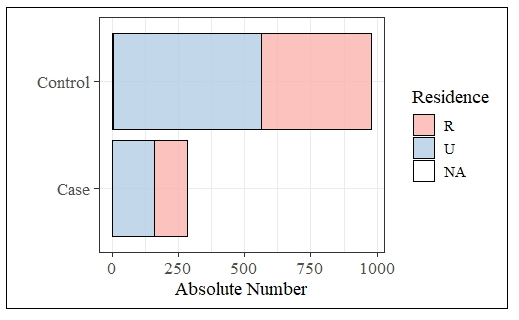


### **Figure 4: Allergy among case- and control patients. 1=yes, 0=no, NA= Not applicable.**


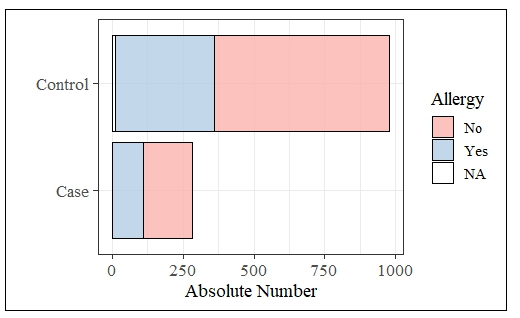


### **Figure 5: Intolerance among case- and control patients. NA= Not applicable.**


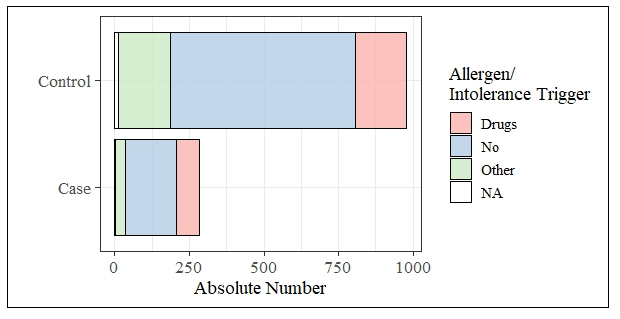


### **Figure 6: Number of drugs at admission among case- and control patients.**


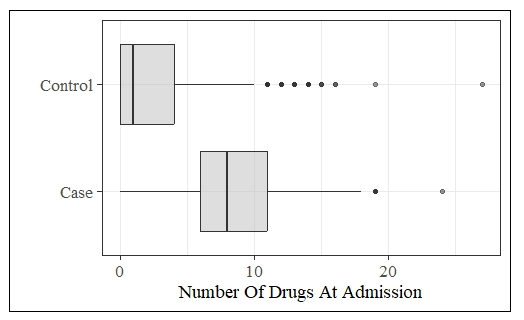


### **Figure 7: American Society of Anesthesiologists (ASA) classification among case- and control patients. NA= Not applicable.**[3]


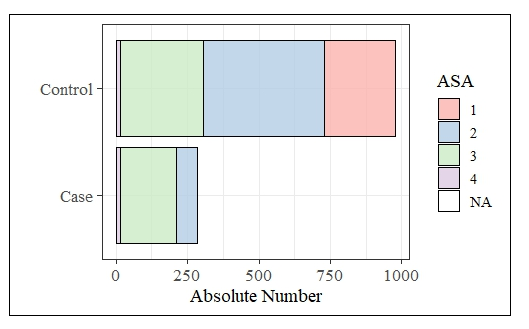


### **Figure 8: Body Mass Index (BMI) among case- and control patients.**


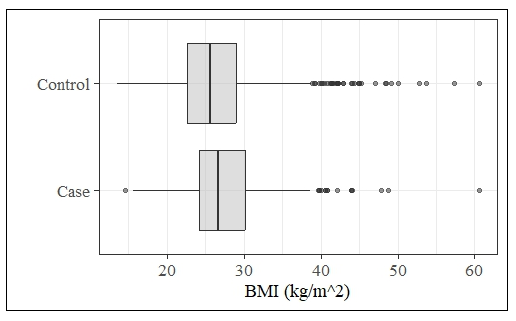


### **Figure 9: Hospitalisation in the last 12 months among case- and control patients. NA=Not applicable.**


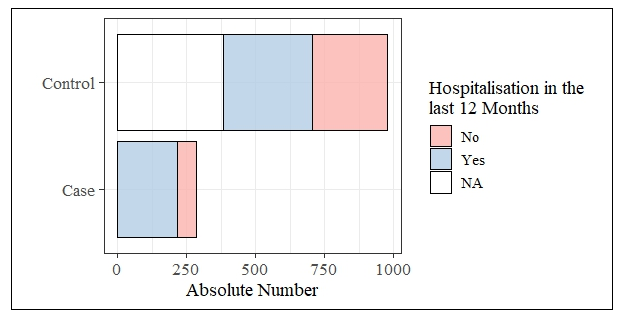


### **Figure 10: Charlson Comorbidity Index (CCI) among case- and control patients.[4]**


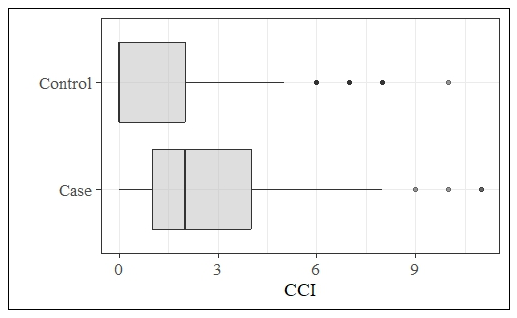


### **Figure 11: Hospital ward among case- and control patients. GF=cardiac surgery, vascular surgery and endovascular surgery, DE=dermatology/allergology, LA=ear, nose and throat diseases, CH=general surgery, GY=gynaecology and obstetrics, KG=oral and maxillofacial surgery, OT=orthopaedics and traumatology, SF=special gynaecology, UR=urology and andrology.**


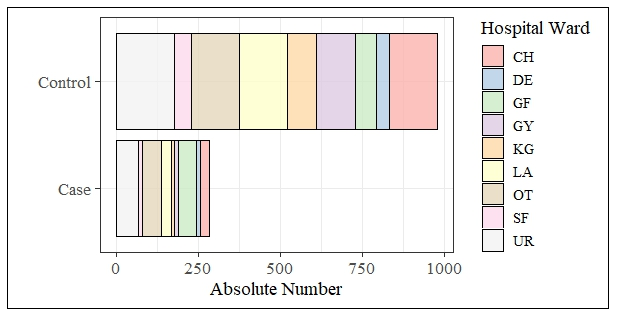


### **Figure 12: Renal function groups among case- and control patients (according to KDIGO 2012).[5]**


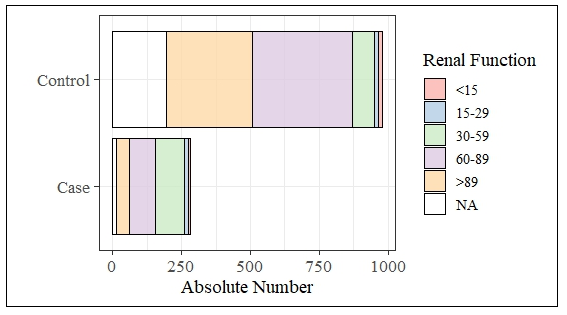


### **References:**

1. Austria S. Zuordnungen - Urban-Rural-Typologie Statistik Austria inkl. Stadtregionen 2022. Available from: <https://www.statistik.at/web_de/klassifikationen/regionale_gliederungen/stadt_land/index.html>.

2. Bundesinstitut für Bau- S-uR. Stadt- und Gemeindetyp 2017. Available from: <https://www.bbsr.bund.de/BBSR/DE/forschung/raumbeobachtung/downloads/downloadsReferenz2.html>.

3. Anesthesiologists ASo. ASA Physical Status Classification System 2020 [1.02.2021]. Available from: <https://www.asahq.org/standards-and-practice-parameters/statement-on-asa-physical-status-classification-system>.

4. Charlson ME, Pompei P, Ales KL, MacKenzie CR. A new method of classifying prognostic comorbidity in longitudinal studies: development and validation. Journal of chronic diseases. 1987;40(5):373-83. Epub 1987/01/01. doi: 10.1016/0021-9681(87)90171-8. PubMed PMID: 3558716.

5. KDIGO. Clinical practice guideline for the evaluation and management of chronic kidney disease. Kidney Int Suppl 2012;3:1-150.
