# Supplementary material for: Development and validation of a risk prediction tool for drug-related problems in pre-operative elective surgical patients (mediPORT): A case-control study
Source: PLoS One. 2025 Sep 2;20(9):e0326088. doi: 10.1371/journal.pone.0326088 (PMC12404507; doi:10.1371/journal.pone.0326088)
Supplement: S2 Table — (DOCX) [file pone.0326088.s004.docx]

### **S2A Table:** Correlations. NO=Number, CCI= Charslon Comorbidity Index.

|  | Age | No of drugs at admission | BMI | Allergy/Intolerance | ASA | Hospitalisation last 12m | CCI |
| --- | --- | --- | --- | --- | --- | --- | --- |
| Age | 1 | 0.54237282 | 0.05055988 | -0.03843261 | 0.61397819 | 0.26863039 | 0.43887573 |
| No of Drugs at Admission | 0.54237282 | 1 | 0.12896961 | 0.0946764 | 0.61118986 | 0.33135276 | 0.43527575 |
| BMI | 0.05055988 | 0.12896961 | 1 | 0.00146303 | 0.11598447 | -0.00508947 | -0.04756342 |
| Allergy/Intolerance | -0.03843261 | 0.0946764 | 0.00146303 | 1 | 0.06996232 | 0.04900743 | 0.02113721 |
| ASA | 0.61397819 | 0.61118986 | 0.11598447 | 0.06996232 | 1 | 0.34051066 | 0.49346787 |
| Hospitalisation last 12 Months | 0.26863039 | 0.33135276 | -0.00508947 | 0.04900743 | 0.34051066 | 1 | 0.26377121 |
| CCI | 0.43887573 | 0.43527575 | -0.04756342 | 0.02113721 | 0.49346787 | 0.26377121 | 1 |

### **S2B Table:** Web code of the variable selection.

| Web code, variable selection | <https://github.com/wandalauth/Project_Mediport.git> |
| --- | --- |
