# Supplementary material for: Development and validation of a risk prediction tool for drug-related problems in pre-operative elective surgical patients (mediPORT): A case-control study
Source: PLoS One. 2025 Sep 2;20(9):e0326088. doi: 10.1371/journal.pone.0326088 (PMC12404507; doi:10.1371/journal.pone.0326088)
Supplement: S3 Table — (DOCX) [file pone.0326088.s005.docx]

### **S3A Table:** Model summary; 5 Variable Model. F=female, NO=Number.

|  | Estimate | Std. Error | z value | Pr(>\|z\|) |
| --- | --- | --- | --- | --- |
| Intercept | -7,297129772 | 0,952956484 | -7,657358855 | 1,89796E-14 |
| Gender F | -0,394421235 | 0,180870001 | -2,180689076 | 0,029206422 |
| Age | 0,042396592 | 0,008831325 | 4,800705671 | 1,58108E-06 |
| No of Drugs at admission | 0,257237146 | 0,023301643 | 11,03944258 | 2,46558E-28 |
| BMI | 0,033992329 | 0,014953728 | 2,273167622 | 0,023016081 |
| Renal Function 15-29 | 0,86186079 | 0,651167586 | 1,32356218 | 0,185648493 |
| Renal Function 30-59 | 1,788806733 | 0,54300073 | 3,294298947 | 0,000986675 |
| Renal Function 60-89 | 0,862978253 | 0,530378965 | 1,627097433 | 0,103716406 |
| Renal Function >89 | 1,26877762 | 0,55560055 | 2,283614765 | 0,022394181 |

### **S3B Table:** Model summary; 2 Variable Model.

|  | Estimate | Std. Error | z value | Pr(>\|z\|) |
| --- | --- | --- | --- | --- |
| Intercept | -5,818405468 | 0,477290985 | -12,19047845 | 3,49364E-34 |
| Age | 0,047677299 | 0,006830625 | 6,979932502 | 2,95322E-12 |
| No of Drugs at admission | 0,26757418 | 0,021606806 | 12,38379142 | 3,19867E-35 |
