# Supplementary material for: Development and validation of a risk prediction tool for drug-related problems in pre-operative elective surgical patients (mediPORT): A case-control study
Source: PLoS One. 2025 Sep 2;20(9):e0326088. doi: 10.1371/journal.pone.0326088 (PMC12404507; doi:10.1371/journal.pone.0326088)
Supplement: S4 Table — (DOCX) [file pone.0326088.s006.docx]

### **S4A Table:** Cut-off values; 5 Variable Model. ROC= Receiver operating characteristic curve.

| Cut-off value | Sensitivity | Specificity | ROC |
| --- | --- | --- | --- |
| 0.01 | 1 | 0.00394203 | 0.8578719 |
| 0.02 | 1 | 0.04342512 | 0.85598808 |
| 0.03 | 0.99696581 | 0.11242029 | 0.8562762 |
| 0.04 | 0.9962963 | 0.17538647 | 0.85684982 |
| 0.05 | 0.99625356 | 0.21222705 | 0.85632845 |
| 0.06 | 0.99625356 | 0.25193237 | 0.8555947 |
| 0.07 | 0.99588319 | 0.27639614 | 0.85701005 |
| 0.08 | 0.99327635 | 0.3071256 | 0.85423323 |
| 0.09 | 0.99250712 | 0.34625604 | 0.85659065 |
| 0.1 | 0.992151 | 0.39045894 | 0.85573739 |
| 0.11 | 0.99065527 | 0.41747826 | 0.85590839 |
| 0.12 | 0.98229345 | 0.4394686 | 0.8573678 |
| 0.13 | 0.97603989 | 0.46448309 | 0.85651693 |
| 0.14 | 0.96183761 | 0.48457005 | 0.85579573 |
| 0.15 | 0.95424501 | 0.5065942 | 0.85697345 |
| 0.16 | 0.94111111 | 0.52602899 | 0.85578348 |
| 0.17 | 0.93373219 | 0.54543478 | 0.85505056 |
| 0.18 | 0.92380342 | 0.55630918 | 0.85407408 |
| 0.19 | 0.91997151 | 0.57669082 | 0.85613723 |
| 0.2 | 0.90794872 | 0.58921739 | 0.85459358 |
| 0.21 | 0.90626781 | 0.60081159 | 0.85719283 |
| 0.22 | 0.8992735 | 0.61610628 | 0.85633914 |
| 0.23 | 0.89535613 | 0.63116908 | 0.85599014 |
| 0.24 | 0.89021368 | 0.64452174 | 0.85583096 |
| 0.25 | 0.8768661 | 0.65874396 | 0.85612214 |
| 0.26 | 0.86868946 | 0.67337198 | 0.85606661 |
| 0.27 | 0.86095442 | 0.68699517 | 0.8564141 |
| 0.28 | 0.8548433 | 0.69917391 | 0.85554599 |
| 0.29 | 0.84839031 | 0.71093237 | 0.85690089 |
| 0.3 | 0.83309117 | 0.72673913 | 0.85667429 |
| 0.31 | 0.82383191 | 0.73608213 | 0.85550084 |
| 0.32 | 0.8125641 | 0.74430435 | 0.85583128 |
| 0.33 | 0.80141026 | 0.75112077 | 0.85743374 |
| 0.34 | 0.78702279 | 0.75867633 | 0.85543659 |
| 0.35 | 0.77653846 | 0.76456522 | 0.85662431 |
| 0.36 | 0.76944444 | 0.77181159 | 0.85639566 |
| 0.37 | 0.75639601 | 0.77770531 | 0.85586375 |
| 0.38 | 0.74574074 | 0.78189372 | 0.85563561 |
| 0.39 | 0.73814815 | 0.78798551 | 0.85623412 |
| 0.4 | 0.7307265 | 0.79304831 | 0.85539018 |
| 0.41 | 0.71849003 | 0.8004058 | 0.85564466 |
| 0.42 | 0.71554131 | 0.80888889 | 0.8559952 |
| 0.43 | 0.70611111 | 0.81129952 | 0.85695734 |
| 0.44 | 0.7004416 | 0.81742995 | 0.85693502 |
| 0.45 | 0.68920228 | 0.82156039 | 0.85506404 |
| 0.46 | 0.67594017 | 0.82265217 | 0.85594632 |
| 0.47 | 0.66623932 | 0.82971014 | 0.85470533 |
| 0.48 | 0.66252137 | 0.83296135 | 0.85664951 |
| 0.49 | 0.65108262 | 0.84104348 | 0.85685643 |
| 0.5 | 0.63854701 | 0.84801449 | 0.85635668 |
| 0.51 | 0.63380342 | 0.851343 | 0.85637017 |
| 0.52 | 0.62109687 | 0.85828502 | 0.85482931 |
| 0.53 | 0.61027066 | 0.86264734 | 0.85649344 |
| 0.54 | 0.60119658 | 0.868343 | 0.85688801 |
| 0.55 | 0.59441595 | 0.87395169 | 0.85551894 |
| 0.56 | 0.58568376 | 0.87627536 | 0.85590261 |
| 0.57 | 0.57653846 | 0.88399517 | 0.85641936 |
| 0.58 | 0.56860399 | 0.88828019 | 0.85775099 |
| 0.59 | 0.56021368 | 0.89215942 | 0.85584834 |
| 0.6 | 0.54653846 | 0.89650242 | 0.85629301 |
| 0.61 | 0.5397151 | 0.90104348 | 0.85660791 |
| 0.62 | 0.53103989 | 0.9054686 | 0.85418424 |
| 0.63 | 0.52384615 | 0.91200483 | 0.85544889 |
| 0.64 | 0.50417379 | 0.91195169 | 0.85640688 |
| 0.65 | 0.49116809 | 0.91986473 | 0.85648965 |
| 0.66 | 0.47511396 | 0.92126087 | 0.85626529 |
| 0.67 | 0.45977208 | 0.9282029 | 0.8561191 |
| 0.68 | 0.44384615 | 0.92950242 | 0.85613566 |
| 0.69 | 0.42988604 | 0.93531884 | 0.85577626 |
| 0.7 | 0.41417379 | 0.93818357 | 0.8564826 |
| 0.71 | 0.39794872 | 0.94060386 | 0.85549532 |
| 0.72 | 0.38178063 | 0.94234783 | 0.85657892 |
| 0.73 | 0.3725641 | 0.94497101 | 0.85604612 |
| 0.74 | 0.35349003 | 0.95044444 | 0.85619983 |
| 0.75 | 0.34354701 | 0.95264734 | 0.85635307 |
| 0.76 | 0.33292023 | 0.95736232 | 0.85670627 |
| 0.77 | 0.32128205 | 0.96224638 | 0.85628095 |
| 0.78 | 0.30588319 | 0.96657005 | 0.85548897 |
| 0.79 | 0.29417379 | 0.97096618 | 0.85773571 |
| 0.8 | 0.28356125 | 0.97558937 | 0.85557043 |
| 0.81 | 0.26696581 | 0.9777343 | 0.85635827 |
| 0.82 | 0.25371795 | 0.97904831 | 0.85470687 |
| 0.83 | 0.24126781 | 0.98011111 | 0.85607107 |
| 0.84 | 0.21935897 | 0.98121256 | 0.85604533 |
| 0.85 | 0.20488604 | 0.98209179 | 0.85613841 |
| 0.86 | 0.19182336 | 0.98255072 | 0.85429642 |
| 0.87 | 0.17625356 | 0.98299517 | 0.85752306 |
| 0.88 | 0.15866097 | 0.98382609 | 0.85643274 |
| 0.89 | 0.14358974 | 0.98496135 | 0.85587644 |
| 0.9 | 0.13004274 | 0.98798068 | 0.85650958 |
| 0.91 | 0.11562678 | 0.98907729 | 0.85625358 |
| 0.92 | 0.10066952 | 0.98974879 | 0.85590317 |
| 0.93 | 0.08477208 | 0.99214493 | 0.85607341 |
| 0.94 | 0.06846154 | 0.99345411 | 0.85706839 |
| 0.95 | 0.05538462 | 0.99344444 | 0.85657862 |
| 0.96 | 0.04519943 | 0.99432367 | 0.8549732 |
| 0.97 | 0.02924501 | 0.99629469 | 0.8563268 |
| 0.98 | 0.01018519 | 0.99782126 | 0.85584671 |
| 0.99 | 0.00112536 | 0.99781643 | 0.8572785 |
| 1 | 0 | 1 | 0.85587299 |

### **S4B Table:** Cut-off values; 2 Variable Model. ROC= Receiver operating characteristic curve.

| Cut-off value | Sensitivity | Specificity | ROC |
| --- | --- | --- | --- |
| 0.01 | 1 | 0 | 0.84682876 |
| 0.02 | 1 | 0.04391304 | 0.84662068 |
| 0.03 | 1 | 0.1161401 | 0.84706019 |
| 0.04 | 1 | 0.15654106 | 0.84654429 |
| 0.05 | 0.9988604 | 0.20111594 | 0.84847211 |
| 0.06 | 0.99623932 | 0.23368116 | 0.84857706 |
| 0.07 | 0.99366097 | 0.26797101 | 0.84775477 |
| 0.08 | 0.99247863 | 0.30079227 | 0.84819519 |
| 0.09 | 0.99247863 | 0.34150725 | 0.848142 |
| 0.1 | 0.98988604 | 0.36819324 | 0.84810597 |
| 0.11 | 0.98048433 | 0.3935942 | 0.84785449 |
| 0.12 | 0.97410256 | 0.41467633 | 0.84832513 |
| 0.13 | 0.9648433 | 0.43576812 | 0.84777545 |
| 0.14 | 0.95876068 | 0.44693237 | 0.84804246 |
| 0.15 | 0.95079772 | 0.46223671 | 0.8487794 |
| 0.16 | 0.94745014 | 0.48164251 | 0.84841046 |
| 0.17 | 0.94737892 | 0.50009662 | 0.84829053 |
| 0.18 | 0.94636752 | 0.52139614 | 0.84902409 |
| 0.19 | 0.9389886 | 0.54345411 | 0.84700665 |
| 0.2 | 0.92809117 | 0.56683092 | 0.84817266 |
| 0.21 | 0.9225641 | 0.58295652 | 0.8485497 |
| 0.22 | 0.90925926 | 0.58941546 | 0.84830201 |
| 0.23 | 0.90433048 | 0.59745894 | 0.84700678 |
| 0.24 | 0.89309117 | 0.60392271 | 0.84666962 |
| 0.25 | 0.88980057 | 0.61527053 | 0.84756936 |
| 0.26 | 0.88160969 | 0.63071981 | 0.84802768 |
| 0.27 | 0.87250712 | 0.64545411 | 0.84793924 |
| 0.28 | 0.86680912 | 0.66432367 | 0.84759457 |
| 0.29 | 0.85742165 | 0.68000483 | 0.84732228 |
| 0.3 | 0.84877493 | 0.69681159 | 0.84785367 |
| 0.31 | 0.84055556 | 0.70847343 | 0.84727447 |
| 0.32 | 0.82611111 | 0.7177343 | 0.84779648 |
| 0.33 | 0.81911681 | 0.729657 | 0.84835813 |
| 0.34 | 0.81421652 | 0.74017391 | 0.8474169 |
| 0.35 | 0.81254986 | 0.74964734 | 0.84734326 |
| 0.36 | 0.80351852 | 0.75676812 | 0.84676503 |
| 0.37 | 0.79746439 | 0.76158937 | 0.84799968 |
| 0.38 | 0.78539886 | 0.76835266 | 0.84863722 |
| 0.39 | 0.77264957 | 0.77888406 | 0.84742309 |
| 0.4 | 0.75974359 | 0.79248792 | 0.84797919 |
| 0.41 | 0.75391738 | 0.80031884 | 0.84870933 |
| 0.42 | 0.7480057 | 0.8067971 | 0.84704676 |
| 0.43 | 0.74121083 | 0.81567633 | 0.84779566 |
| 0.44 | 0.73452991 | 0.81971014 | 0.84784729 |
| 0.45 | 0.72052707 | 0.82616425 | 0.84732943 |
| 0.46 | 0.7027208 | 0.83139614 | 0.8465002 |
| 0.47 | 0.68997151 | 0.83926087 | 0.8475586 |
| 0.48 | 0.67445869 | 0.84288889 | 0.84828528 |
| 0.49 | 0.66334758 | 0.84627536 | 0.84828959 |
| 0.5 | 0.63820513 | 0.85131401 | 0.84767185 |
| 0.51 | 0.62031339 | 0.85363285 | 0.84717922 |
| 0.52 | 0.61183761 | 0.8555942 | 0.84868046 |
| 0.53 | 0.6008547 | 0.86065217 | 0.84642036 |
| 0.54 | 0.58827635 | 0.86530435 | 0.84646358 |
| 0.55 | 0.56904558 | 0.86763768 | 0.84743464 |
| 0.56 | 0.55722222 | 0.87250242 | 0.8479854 |
| 0.57 | 0.54430199 | 0.87575845 | 0.84744577 |
| 0.58 | 0.53247863 | 0.87966184 | 0.84720807 |
| 0.59 | 0.51578348 | 0.88594203 | 0.84803035 |
| 0.6 | 0.502151 | 0.89274879 | 0.84821623 |
| 0.61 | 0.49049858 | 0.90128986 | 0.84777514 |
| 0.62 | 0.48223647 | 0.90526087 | 0.84805044 |
| 0.63 | 0.46616809 | 0.90871981 | 0.84733788 |
| 0.64 | 0.44700855 | 0.91264734 | 0.847793 |
| 0.65 | 0.43287749 | 0.91503382 | 0.84823446 |
| 0.66 | 0.41152422 | 0.91898551 | 0.84781923 |
| 0.67 | 0.39900285 | 0.92227536 | 0.84876685 |
| 0.68 | 0.38320513 | 0.925343 | 0.84853136 |
| 0.69 | 0.36897436 | 0.9305942 | 0.8468726 |
| 0.7 | 0.35745014 | 0.93604348 | 0.84785523 |
| 0.71 | 0.33547009 | 0.93862802 | 0.84774798 |
| 0.72 | 0.31584046 | 0.94255072 | 0.84785461 |
| 0.73 | 0.30071225 | 0.94389372 | 0.8481566 |
| 0.74 | 0.28327635 | 0.94850242 | 0.84766585 |
| 0.75 | 0.2655698 | 0.9495942 | 0.84716285 |
| 0.76 | 0.24988604 | 0.95589372 | 0.84711599 |
| 0.77 | 0.24310541 | 0.95910628 | 0.84854707 |
| 0.78 | 0.23042735 | 0.96223671 | 0.84881754 |
| 0.79 | 0.22292023 | 0.96352174 | 0.84690543 |
| 0.8 | 0.21287749 | 0.96720773 | 0.84774293 |
| 0.81 | 0.19931624 | 0.97009179 | 0.84731617 |
| 0.82 | 0.18552707 | 0.97358937 | 0.84907182 |
| 0.83 | 0.1735755 | 0.97596618 | 0.84804285 |
| 0.84 | 0.15464387 | 0.97947826 | 0.84829914 |
| 0.85 | 0.14374644 | 0.98139614 | 0.84783376 |
| 0.86 | 0.13589744 | 0.98452657 | 0.84759194 |
| 0.87 | 0.12623932 | 0.9871256 | 0.84745009 |
| 0.88 | 0.11545584 | 0.99062319 | 0.84762278 |
| 0.89 | 0.10521368 | 0.99104348 | 0.84800996 |
| 0.9 | 0.08363248 | 0.99170048 | 0.84774447 |
| 0.91 | 0.06698006 | 0.99475362 | 0.84823784 |
| 0.92 | 0.05331909 | 0.99562802 | 0.84703846 |
| 0.93 | 0.04646724 | 0.99561353 | 0.84820136 |
| 0.94 | 0.03933048 | 0.99564251 | 0.84876421 |
| 0.95 | 0.02673789 | 0.99563285 | 0.84751117 |
| 0.96 | 0.01309117 | 0.99563768 | 0.84808961 |
| 0.97 | 0.01012821 | 0.99562319 | 0.84661576 |
| 0.98 | 0.00447293 | 0.99738164 | 0.84763171 |
| 0.99 | 0.00111111 | 0.99782126 | 0.84772521 |
| 1 | 0 | 1 | 0.84806468 |
