# Supplementary material for: Development and validation of a risk prediction tool for drug-related problems in pre-operative elective surgical patients (mediPORT): A case-control study
Source: PLoS One. 2025 Sep 2;20(9):e0326088. doi: 10.1371/journal.pone.0326088 (PMC12404507; doi:10.1371/journal.pone.0326088)
Supplement: Attachment — Submitted filename: Response to Reviewers.pdf [file pone.0326088.s008.pdf]

PARACELSUS
MEDICAL
UNIVERSITY

Dear Editor, dear Reviewers,

we sincerely appreciate your valuable insights and the time you dedicated to reviewing our manuscript. Your suggestions have significantly enhanced its quality. Following your recommendations, we have revised the manuscript accordingly and provided a detailed, point-by-point response. Our replies are in italics, and the revisions to the manuscript are highlighted in blue. For some of the suggestions, we would like to clarify the following:

- We have shortened the abstract
- We have updated references to include more recent sources and have gone from 35 to 39 references
- We have extent the introduction
- A language check was conducted by a natural speaker

We hope that these changes to the manuscript will meet your expectations.

On behalf of the authors,

Sincerely,

Stephanie Clemens

Reviewer #1:

1. **Too long abstract. You can shorten it.**
   ➔ *Thank you the review and for your helpful suggestion. We have shortened the abstract, reducing it from 299 words to 264 words.*

2. **Too many old ref. you can use the recent ref (within the last 5 years).**
   Please use (google scholar and Refseek) search engines then set it since 2020
   you can use my suggestions
   ➔ *Thank you for this valuable suggestion and the method for identifying more recent studies. We have applied this approach and updated the majority of the references, replacing older studies with newer ones and revising the text to align with the content of the updated studies.*

Early identification of vulnerable patients can improve medication safety, quality of care and can lead to substantial cost reductions for the healthcare system [6].

New reference:
[6] Falconer N, Barras M, Abdel-Hafez A, Radburn S, Cottrell N. Development and validation of the Adverse Inpatient Medication Event model (AIME). Br J Clin Pharmacol. 2021;87(3):1512-24. Epub 2020/09/29. doi: 10.1111/bcp.14560. PubMed PMID: 32986855.

Recently published systematic reviews on prediction tools of DRP highlight the increasing interest in patient prioritization. The primary challenges with existing predictive tools include their moderate performance, limited validation and difficulty in adapting to diverse clinical settings. Additionally, there is a notable absence of straightforward, easily accessible variables that can be quickly utilized without relying on complex assessment frameworks [7, 8].

New references:
[7] Deawjaroen K, Sillabutra J, Poolsup N, Stewart D, Suksomboon N. Clinical usefulness of prediction tools to identify adult hospitalized patients at risk of drug-related problems: A systematic review of clinical prediction models and risk assessment tools. Br J Clin Pharmacol. 2022;88(4):1613-29.

[8] Jung-Poppe L, Nicolaus HF, Roggenhofer A, Altenbuchner A, Dormann H, Pfistermeister B, et al. Systematic Review of Risk Factors Assessed in Predictive Scoring Tools for Drug-Related Problems in Inpatients. J Clin Med. 2022;11(17).

Furthermore, research on risk factors, which are associated with DRP is scarce for surgical patients. Studies have evaluated outcomes of ADE and adverse drug reactions (ADR) in hospitalized patients [6, 9, 10].

New references:
[6] Falconer N, Barras M, Abdel-Hafez A, Radburn S, Cottrell N. Development and validation of the Adverse Inpatient Medication Event model (AIME). Br J Clin Pharmacol. 2021;87(3):1512-24.

[10] Lima S, Martins RR, Saldanha V, Silbiger VN, Dos Santos ICC, Araujo IB, et al. Development and validation of a clinical instrument to predict risk of an adverse drug reactions in hospitalized patients. PloS one. 2020;15(12):e0243714.

Saldanha et al. and Lima et al. developed an internally validated risk score on the various types of DRP in adult patients admitted to a general hospital [10, 11].

New references:
[10] Lima S, Martins RR, Saldanha V, Silbiger VN, Dos Santos ICC, Araujo IB, et al. Development and validation of a clinical instrument to predict risk of an adverse drug reactions in hospitalized patients. PloS one. 2020;15(12):e0243714.

[11] Saldanha V, Araujo IB, Lima S, Martins RR, Oliveira AG. Risk factors for drug-related problems in a general hospital: A large prospective cohort. PloS one. 2020;15(5):e0230215.

Most studies assessed risk factors in older adults [4, 13] or medical specialties such as internal medicine [9, 14]

New references:
[4] Brady A, Curtis CE, Jalal Z. Screening Tools Used by Clinical Pharmacists to Identify Elderly Patients at Risk of Drug-Related Problems on Hospital Admission: A Systematic Review. Pharmacy (Basel). 2020;8(2).

[13] Puumalainen E, Airaksinen M, Jalava SE, Chen TF, Dimitrow M. Comparison of drug-related problem risk assessment tools for older adults: a systematic review. Eur J Clin Pharmacol. 2020;76(3):337-48

[9] Botelho SF, Neiva Pantuzza LL, Marinho CP, Moreira Reis AM. Prognostic prediction models and clinical tools based on consensus to support patient prioritization for clinical pharmacy services in hospitals: A scoping review. Research in Social and Administrative Pharmacy. 2021;17(4):653-63. doi: https://doi.org/10.1016/j.sapharm.2020.08.002.

[14] Audurier Y, Roubille C, Manna F, Zerkowski L, Faucanie M, Macioce V, et al. Development and validation of a score to assess risk of medication errors detected during medication reconciliation process at admission in internal medicine unit: SCOREM study. International journal of clinical practice. 2021;75(2):e13663.

Stewart et al. and Rose et al. identified patient factors, predicting a high benefit from medication reviews in the primary care setting [15].

New reference:
[15] Stewart AE, Lovato JF, Zimmer R, Stewart AP, Hinely MT, Yang M. Development of a screening tool to identify patients likely to benefit from clinical pharmacist review in a home-based primary care population. J Am Pharm Assoc. 2020;60(5):750-6.

Initially, the ratio of controls to cases was set to 4:1 as statistical power strongly increases within this ratio [21] and the prevalence of DRP in the target population was low [22].

New reference:
[22] Simon C, Rose O, Kanduth K, Pachmayr J, Clemens S. Drug-related problems in elective surgical inpatients: A retrospective study. Science Progress. 2024;107(3) 1–15. doi: 10.1177/00368504241263534.

Previous developed models showed reasonable specificity but lacked in sensitivity, which was lower than 62% [10,31].

New references:
[10] Lima S, Martins RR, Saldanha V, Silbiger VN, Dos Santos ICC, Araujo IB, et al. Development and validation of a clinical instrument to predict risk of an adverse drug reactions in

hospitalized patients. PloS one. 2020;15(12):e0243714. Epub 2020/12/12. doi: 10.1371/journal.pone.0243714. PubMed PMID: 33306728; PubMed Central PMCID: PMCPMC7732084.

[29] Hoj K, Pedersen HS, Lundberg ASB, Bro F, Nielsen LP, Saedder EA. External validation of the Medication Risk Score in polypharmacy patients in general practice: A tool for prioritizing patients at greatest risk of potential drug-related problems. Basic Clin Pharmacol Toxicol. 2021;129(4):319-31.

Falconer et al. did not find any risks associated with renal function [6].

New reference:
[6] Falconer N, Barras M, Abdel-Hafez A, Radburn S, Cottrell N. Development and validation of the Adverse Inpatient Medication Event model (AIME). Br J Clin Pharmacol. 2021;87(3):1512-24.

Reviewer #2:

1. **Thank you so much for your exceptional work and dedication in addressing all the comments provided by the previous reviewers. Your thorough and thoughtful approach to incorporating the feedback has undoubtedly enhanced the overall quality and clarity of the work. It is clear that you have invested significant effort into ensuring that every aspect of the reviewers' suggestions has been considered and addressed comprehensively.**
However, I suggest that to make a plan to validate the prediction model in more hospitals with different study design.
   ➔ *Thank you very much for your thoughtful feedback and kind words regarding our dedication to addressing the reviewers' comments. We greatly appreciate your suggestion to validate the prediction model in more hospitals with different study designs. This is something we are actively working on, and to reflect this in the manuscript, we have added the following statement:*

   Currently, external validation of the tool across various settings in Austria is planned.

   We are committed to further enhancing the model's robustness through this validation.

Reviewer #3:

1. **Most research aspects are performed perfectly. The research idea is novel. The objectives were clarified and listed. The methodology chosen and applied in good way and was sufficient to explore and answer the research questions and achieving the research objectives. The findings showed clearly in the result section. The study findings and thoughts discussed optimally.**
   ➔ *Thank you for the review and for your kind feedback on our study; we appreciate your recognition of the research idea, methodology and clarity of the findings.*

2. **I suggest to expand the introduction section to cover the relevant literature adequately. In addition, proofreading for the full manuscript is required to address few grammatical mistakes existing within the submitted manuscript.**
No further comments is needed. I endorse publication with minor amendments.
Best wishes.
   ➔ *Thank you for your valuable suggestion. We have expanded the introduction to incorporate the two most recent systematic reviews, highlighting current challenges, key*

study findings, and cost reduction aspects. Additionally, we have thoroughly proofread the manuscript to address any grammatical issues.

Early identification of vulnerable patients can improve medication safety, quality of care and can lead to substantial cost reductions for the healthcare system [6]. Recently published systematic reviews on prediction tools of DRP highlight the increasing interest in patient prioritization. The primary challenges with existing predictive tools include their moderate performance, limited validation and difficulty in adapting to diverse clinical settings. Additionally, there is a notable absence of straightforward, easily accessible variables that can be quickly utilized without relying on complex assessment frameworks [7, 8].

According to Saldanha et al., several risk factors for DRP were identified based on patient variables at admission and drugs prescribed during the first two days of hospitalization. Key factors included an elevated heart rate and the use of multiple drugs targeting the alimentary/metabolic system, systemic anti-infectives or drugs affecting blood or its components. Patients admitted for elective surgery had a reduced risk of DRP [11].

In this study, thirteen risk factors for DRP were identified including socioeconomic status, comorbidity and medication count, estimated glomerular filtration rate, white cell count, allergies, specific drug classes, and diagnoses. These factors were incorporated into an Excel-based tool that predicts the risk for DRP and classifies patients into three different risk categories [12].

Key factors that influenced the effectiveness of medication reviews included the number of drugs, discrepancies between prescribed and actual usage, baseline Medication Appropriateness Index (MAI), and the duration of the intervention. While most of these parameters required extensive patient assessment, the number of drugs were administrable for an initial screening [16]. In a follow-up study, Rose et al. demonstrated that physicians' intuitive judgments served as a reasonably effective predictive factor. However, these judgments should be surpassed by any screening tool considered the gold standard [17].

The resulting tool, a self-administered questionnaire, was validated to classify hospitalized non-acute older patients based on their risk for DRP [19].
