# Supplementary material for: Development and validation of a risk prediction tool for drug-related problems in pre-operative elective surgical patients (mediPORT): A case-control study
Source: PLoS One. 2025 Sep 2;20(9):e0326088. doi: 10.1371/journal.pone.0326088 (PMC12404507; doi:10.1371/journal.pone.0326088)
Supplement: Attachment — Submitted filename: Response_to_Reviewers_auresp_2.pdf [file pone.0326088.s009.pdf]

PARACELSUS MEDICAL UNIVERSITY

**Dear Editor, dear Reviewers,**

we sincerely appreciate your valuable insights and the time you dedicated to reviewing our revised manuscript. Your suggestions have significantly enhanced its quality. Following your recommendations, we have revised the manuscript accordingly and provided a detailed, point-by-point response. Our replies are in italics, and the revisions to the manuscript are highlighted in blue. For the suggestions, we would like to clarify the following:

- We have revised Figure 4
- We have provided a more detailed explanation of the rationale behind choosing a 1:4 case-control ratio
- We have added a statement to the discussion highlighting the potential for future analyses involving drug class stratification

We hope that these changes to the manuscript will meet your expectations.

On behalf of the authors,

Sincerely,

Stephanie Clemens

**Reviewer #4:**

**The study was great and would have significant contributions to the pharmacy practices.**
**The authors have responded to the previous reviewer's comments. Most of the comments and research aspects were described clearly.**
**However, there is an additional minor comment from the file PONE-D-24-34407_R1_reviewer as follows:**

1. **Please describe in more detail the rationale for setting the sample case-control ratio of 1:4 (line 181 or 149 updated version)**

*Thank you for your kind words about our study and for this important and constructive comment. The prevalence of drug-related problems (DRPs) in our target population was relatively low, with 9.89% (n = 352) of patients experiencing at least one DRP (mean 1.43, SD 0.7) [1]. Based on the limited number of cases, we selected a 1:4 case-control ratio. We have revised the manuscript to provide a more detailed rationale for this choice, and have added the following:*

Given this relatively low prevalence, it was anticipated in the study planning that the number of eligible cases would be limited. Therefore, a 1:4 case-control ratio was selected not only for statistical efficiency but also to ensure a sufficient total sample size to power the analysis. This ratio allowed for better estimation of exposure-outcome associations and model parameters without the need to include all available cases.

*This rationale is now connected to Section "Population" and "Sample size," where we explain that*
cases and controls were sampled from a single source population (n=11,176) between January 1, 2021 (01.01.2021), and December 31, 2021. To reach the target case-control ratio of 1:4, a random selection of 1200 patient controls was carried out. Consequently, the target sample size (i.e., including cases and controls) was set to 1500 patients. This sample size allowed to estimate the sensitivity of the multivariable model to a precision of plus/minus 5% in terms of the width of a 95% CI, under the assumption that the sensitivity was at least 75%. Moreover, the number of cases was also adequate with respect to the generally recommended minimum event-per-variable (EPV) ratio of 10 [20].

*Furthermore, the rationale is now underpinned by the number of cases and controls in the section*
During the year 2021, there were 11,176 admissions for elective surgery. Of these, 1200 patients without a documented DRP were selected at random and 300 patients with a DRP, meeting the inclusion criteria, were included. Patients not reviewed by clinical pharmacists (e.g., day care surgery or <18 years) were excluded. The full participant flow is shown in Fig 3. Given the final sample size and number of predictors (12 variables), the anticipated EPV was 25, exceeding the commonly accepted threshold. Even after splitting the data into training and test sets (70%/30%), the EPV in the training sample remained well above 10, thereby reducing the risk of overfitting and underfitting the model [20].

*Thank you again for your revision suggestion. We hope that it adequately addresses your concern and improves the transparency of our study design.*

**Reviewer #5:**

**I would like to sincerely thank the authors for their thoughtful and rigorous work on this important topic. The manuscript reflects a high level of scholarly effort, methodological care, and clinical insight. The development of the mediPORT tool represents a meaningful contribution to improving medication safety in surgical care, and I commend the authors for their dedication to advancing patient-centered clinical pharmacy practice.**
**Strengths:**

**• The study addresses a significant gap in perioperative pharmacotherapy and patient safety.**
**• The use of a large, well-defined dataset and robust statistical modeling (including internal validation) enhances the credibility of the findings.**
**• The inclusion of both a comprehensive and a simplified model increases the tool's applicability across diverse clinical settings.**
**Recommendations for Minor Revision:**
**The observed higher DRP risk in CKD stage 3 compared to stage 5 warrants further discussion. The authors may consider elaborating on potential clinical explanations or stratifying by drug class in future analyses.**

*Thank you for your kind words regarding our study and for your valuable comment. We have incorporated the important aspect of drug class stratification in future analyses, which will help to improve risk stratification and guide clinical decision-making.*

Future analyses stratified by drug class or pharmacologic group could provide greater insight into which medications drive DRP risk in moderate versus severe CKD. Understanding these nuances may ultimately improve risk stratification and prevention strategies in clinical practice.

**• Ensure all figures are high-resolution and clearly labeled. Figure 4 (mediPORT tool) could benefit from a more intuitive visual layout**

*Thank you for your valuable feedback. We have ensured that all figures are provided in high resolution (600dpi) and checked the labels. Additionally, we have redesigned Figure 4 to feature a more intuitive visual layout to enhance readability and comprehension.*

***Before:***

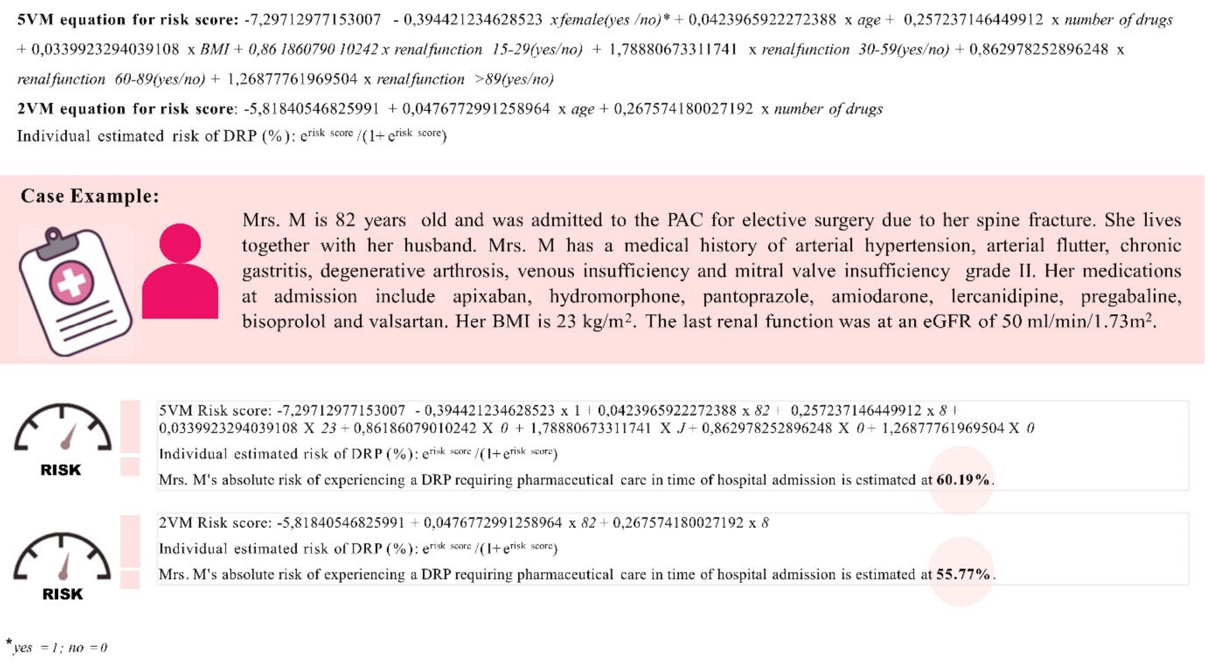

***After:***

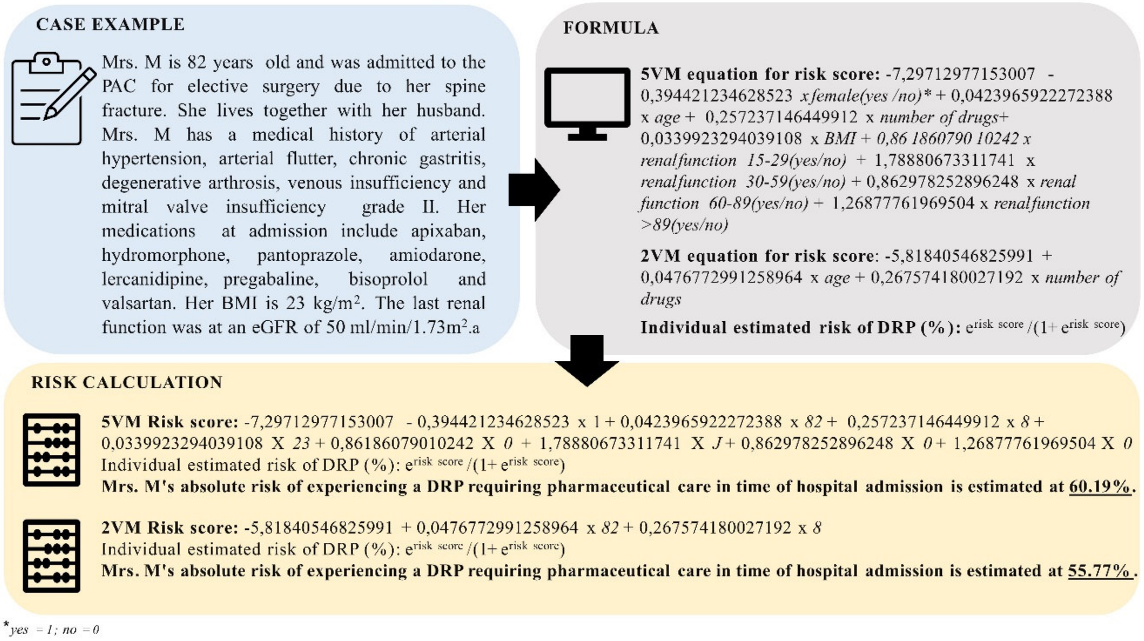


1.

Fig 4. MediPORT tool. Calculation of patient risk for developing a drug-related problem requiring pharmaceutical care at hospital admission. 5VM: 5 Variable Model, 2VM: 2 Variable Model, DRP: drug-related problem, PAC: pre-anesthesia clinic, BMI: body mass index, eGFR: estimated glomerular filtration rate.

**References:**

Simon C, Rose O, Kanduth K, Pachmayr J, Clemens S. Drug-related problems in elective surgical inpatients: A retrospective study. Science Progress. 2024;107(3) 1–15. doi: 10.1177/00368504241263534.
