# Supplementary material for: Development and validation of a risk prediction tool for drug-related problems in pre-operative elective surgical patients (mediPORT): A case-control study
Source: PLoS One. 2025 Sep 2;20(9):e0326088. doi: 10.1371/journal.pone.0326088 (PMC12404507; doi:10.1371/journal.pone.0326088)
Supplement: Attachment — Submitted filename: Response to Editor and Reviewers_SC_2024.docx [file pone.0326088.s007.docx]

*Response to Reviewers letter including a point by point response to each of the comments of our previous decision letters at 12th June 2024 (major review) and 31st July 2024 (rejection).*

# 12^th^ June 2024 (major review):

**Dear Editor, dear Reviewers,**

Thank you very much for your expertise and time. We appreciate your suggestions, which have greatly contributed to improving the quality of our manuscript. In accordance with your advice, we have made amendments to the manuscript and provided a point-by-point response. Our responses are indicated in italics, and changes to the manuscript are highlighted in blue. Regarding some suggestions, we would like to note the following:

- We wish to highlight the invaluable contribution of two statisticians from IDA Lab Salzburg who played an active role in the study’s statistical analysis. Their efforts encompassed meticulous planning of variable selection, sample size calculation, careful consideration of model uncertainty, and thorough model validation. Furthermore, we have taken great care to address reviewer comments, particularly those pertaining to unclear wording, to ensure that the revised sections are both clear and accurate.
- A language check was conducted by a natural speaker
- We revised one affiliation (of the author GZ) due to a change of institution
- We have revised Figure 1 and Figure 5 to enhance clarity
- We have added information on cut-off values in the supplementary data (S4 Table. Cut-off values of the model)
- We have gone from 29 to 34 references

We hope that these changes to the manuscript will meet your expectations.

On behalf of the authors,

Sincerely,

Stephanie Clemens

**Reviewer #1:**

1. **Line 47 The study is a retrospective, single centre, case-control, observational study…I believe the authors can use just ‘case-control’ because all case control studies are observational and retrospective so no need to make redundancies.**

- *Thank you for the relevant comment. We revised this aspect:*

Elective surgery patients aged ≥ 18 years admitted to pre-anaesthesia clinic and participating in a medication review by pharmacists were included in this case-control study.

2. **Line 50 Routinely reported patient variables were involved in a stepwise backwards elimination regression model…What type of regression model?**

- *Thank you for this comment. We specified on the used regression model:*

Routinely reported patient variables were included in a multivariable logistic regression model with backward stepwise selection to determine the most relevant predictors (minimum Akaike Information Criterion) of DRP.

3. **Line 54 Is 11,176 your study population or target population?**

- *Thank you for the relevant hint. We revised this sentence:*

The target population consisted of 11,176 participants, of whom 284 cases with ≥ 1 DRP and 980 controls without DRP were drawn.

4. **Line 50& line 55, the authors should explain what they mean by strong or strongest predictors? Did they also identify weak predictors?**

- *Thank you for your valuable comment on this aspect. We used multivariable logistic regression with backward stepwise selection and ranked the set of variables based on the value of the Akaike Information Criterion (AIC). In the context of our specific data, the variable set exhibiting the lowest AIC value was considered the most suitable among the available models. Due to the fact that we used AIC-based backward selection, the finally included variables in the model are also (highly) significant, as reported in the subsection “Development of the mediPORT risk model”. Based on your comment, we revised the definition of the term and changed it into “in most relevant predictors” (in the entire manuscript) and described its meaning:*

Routinely reported patient variables were included in multivariable logistic regression with backward stepwise selection to determine the most relevant predictors (minimum Akaike Information Criterion) of DRP.

5. **Line 64&65: May the authors justify that with sensitivity/specificity <80%, are the current tools clinically applicable? i.e. missing an actual DRP in >20% of the patients.**

- *The screening tool demonstrates sensitivities of 77.6% (5VM) and 81.3% (2VM), along with specificities of 76.5% (5VM) and 75% (2VM), enabling effective pre-surgical assessment of surgical inpatients for potential DRP. These values surpass those documented in other studies focused on the development and validation of risk-prediction scores for DRP [1-4]. Nonetheless, it is important to acknowledge that the tool may overlook certain DRP and should therefore be utilized with caution. We have added the aspects to the manuscript (Abstract and Discussion).*

Findings of this study complement previous research on priority setting. Resulting equations can be used by hospital admission, to identify patients at high risk, for whom a precise assessment of medication is critical.

In modern healthcare, it is essential to base the practical use of tools on the best available evidence. This means applying evidence on test performance with careful judgment and testing efficiently to optimize patient care. Moreover, considering the value and affordability of a test before requesting it ensure resources are used effectively. It is also crucial to be aware of the downsides and drivers of over diagnosis, confront uncertainties, and maintain a patient-centred approach. Addressing ethical issues and recognizing cognitive biases further enhances the quality of testing. Teaching and learning these core principles through a systematic approach ensures that healthcare professionals can navigate the complexities of testing with confidence and precision [5]. When using the mediPORT tool, clinical pharmacists can intervene during medication reconciliation and patient interviews at the PAC visit to rectify any omissions.

6. **Line 75 one approach to increase the health and care workforce…. health and care workforce should be rephrased.**

- *Thank you for the comment. We revised this sentence:*

According to the World Health Organization (WHO), one approach to enhance healthcare capacity is to expand the use of digital tools.

7. **Line 76 pharmaceutical service is too general.**

- *Thank you for the important hint. We specified the pharmaceutical service in terms of medication management:*

This activity is time-consuming, hence personnel shortages and limited resources impose a noteworthy barrier to clinical pharmacy interventions in medication management.

8. **Line 85-92… needs extensive rephrasing using objective and clear statements. Too long statements should be rewritten in to short and clear sentences.**

- *Thank you for this valuable comment. We totally agree, and revised the paragraph:*

Another study selected risk factors for DRP based on expert opinions [6]. Developing tools to prioritize patients for pharmacotherapy optimization is complex. It faces barriers such as method selection, predictor choice, cut-off point estimations, and validation [7]. The present study endeavors to fill a knowledge gap in available tools by developing and validating a diagnostic model.

9. **Line 93. potential and manifest DRP. Which one was included in the prediction model? What is the clinical applicability of predicting potential DRP? Prediction by itself is not 100% sensitive; again, if you predict some event that is potential, it is clinically not applicable. Please explain.**

- *In our prediction model, both types of DRP were included. It is important to note that predicting potential DRP may not always translate directly into clinical applicability. A DRP is defined as an event or circumstance involving drug therapy that actually or potentially interferes with desired health outcomes [8]. While predicting potential DRP provides valuable insights, it is essential to consider the clinical context and the likelihood of the predicted event actually occurring. Based on your relevant comment, we added information to the manuscript including an example on clinical applicability:*

A DRP is defined as “*an event or circumstance involving drug therapy that actually or potentially interferes with desired health outcomes*”[8].

This model aims to identify high-risk elective surgical patients being prone for experiencing potential and manifest DRP, which need to be reviewed more closely. The clinical value of such a DRP prediction tool is to highlight patients at risk, so that caution can be taken and DRP can be resolved, before they become significant.

While predicting potential DRP provides valuable insights, it is essential to consider the clinical context and the likelihood of the predicted event to actually occur. For example, even if a patient is predicted to experience a potential DRP, such as requiring a dosage adjustment for a drug due to renal insufficiency, it may not be clinically relevant in this specific case. While diagnostic models can inform clinical decision-making, their application must be considered in conjunction with the clinical context and patient-specific factors.

10. **most significant risk factors. Did the authors also identify less significant factors? Were these included?**

- *Thank you for the important hint. According to your comments 4 and 16, we revised the terminology to most relevant predictors based on the minimum AIC as a result of the multivariable logistic regression with backward stepwise selection. Additionally, we repeated the splitting into training and test sets as well as the respective variable selection process 1000 times, in order to reflect the uncertainty in the selection process, and include only those variables into the final model that were selected in at least 50% of the runs (for details, see the “Model development” subsection). Therefore, we agree with you that “significant” in this case is a misleading term. It erroneously refers to the significance tests (cases vs. controls) but does not go hand in hand with the model development by multivariate logistic regression using the AIC. Furthermore, we also clarified that the term “variables” is synonym with “predictors” in this manuscript:*

Study objectives were to: a) identify predictors for potential and manifest DRP in elective surgical patients, b) develop a multivariate score based on most relevant predictors, to anticipate the risk of existing DRP and c) validate the tool in the remaining sample.

The following factors were applied when determining the final set of variables (predictors)…

11. **Line 90-91: fill a knowledge gap regarding development and validation of a diagnostic model to identify high-risk elective surgical patients….I there any gap of diagnostic model development process itself? What do the authors mean by high-risk elective surgical patients? Does this have anything to do with DRP?**

- *Thank you for your comment. We agree, there are some issues in readability and spelling. The term "high-risk" suggests that these patients may be more prone to experiencing medication-related issues during the perioperative period. Therefore, identifying such patients is crucial for preemptively managing and mitigating potential DRP to ensure optimal patient outcomes. We revised this sentence to clarify:*

The present study endeavors to fill a knowledge gap in available tools by developing and validating a diagnostic model. This model aims to identify high-risk elective surgical patients being prone for experiencing potential and manifest DRP, which need to be reviewed more closely.

**Methods**

12. **Line 116. The study design was a retrospective, observational, single-center, unmatched population-based case- control study….as commented above in abstract**

- *Thank you for this comment. We totally agree and revised this sentence:*

The study design was a case-control study (Fig 1).

13. **The authors should justify if case control design is ideal to develop a risk prediction model. The concern is if all DRPs were identified and documented. What if the patients actually had DRPs but were not assessed by clinical pharmacists which is common scenario because of the shortage of clinical pharmacists or lack of risk prioritization methods. It should come out clear if all the controls had also been reviewed by clinical pharmacists or not.**

- *Thank you for the important hint. We totally agree on clarifying this aspect. Pharmacists reviewed all included cases. Patients with no review of clinical pharmacists (i.e. day care surgery patients and patients under the age of 18 years) were excluded. Numbers of excluded patients were displayed in the patient flow chart diagram (Fig 3). We clarified the aspect in the manuscript:*

Controls, all of whom were reviewed by clinical pharmacists, were defined as elective surgical patients without any identified and documented DRP.

14. **Line 123. controls to cases was set to 4:1. Based on which previous prevalence or what other reference was used?**

- *We fully concur with this perspective. Woodward et al. (2013) highlighted that in unmatched case-control settings, statistical power significantly rises up to a ratio of 4:1 or 5:1, followed by a gradual increase thereafter [9]. Considering the low prevalence of DRP in the target population and the observed increase in statistical power at ratios of 4:1, we have added the following information:*

Initially, the ratio of controls to cases was set to 4:1 as statistical power strongly increases within this ratio [9, 10] and the prevalence of DRP in the target population was low.

15. **Line 152-160. It is not clear if sample size calculation was used or size was determined according to availability of data; they are contradicting. Sample size should have been calculated for the specific predictors separately during the development study. Sample size for validation study should be clearly discussed separately.**

- *We totally agree, thank you for the important hint. We deleted the misleading sentence “The sample size was determined according to the availability of existing data” to enhance clarity.*

According to a population-based case-control study design, cases and controls were sampled from a single source population (n=11,176) between 1st January and 31st December 2021. To reach the target case-control ratio of 1:4, a random selection of 1200 patient controls was carried out. Consequently, the target sample size (i.e., including cases and controls) was set to 1500 patients. This sample size allowed to estimate the sensitivity of the multivariable model to a precision of plus/minus 5% in terms of the width of a 95% CI, under the assumption that the sensitivity was at least 75%. Moreover, the number of cases was also adequate with respect to the generally recommended minimum event-per-variable (EPV) ratio of 10 [11].

16. **Line 161. Predictors or risk factors? The authors need to be consistent throughout the document.4**

- *We totally agree on this aspect. According to the comments (no. 4 , 10) we consistently use the term “predictor”.*

**Results**

17. **It should be clearly discussed how the models are scored. What cut off points are the authors referring to when they discuss the sensitivities and specificities? If these are not clear, how do practitioners be able to score and use?**

- *Thank you for the very important hint. We included all cut-off values in a further appendix (Table S3) to make it more clearly for practitioners and added the following information to the manuscript:*

The range of cut-off values for both models is provided in the S4 Table. All results for the mediPORT tool are based on the chosen cut-off value of 0.35 for both models.

- *Furthermore, we clarified this aspect by adding the information to the discussion:*

Choosing an optimal cut-off value is challenging as a model with higher sensitivity will report lower specificity and vice versa. While patients identified as false positive (type I error) may result in increased medication scrutiny, the information on risk has potential to cause unnecessary concern to patients and clinicians. Additionally, unnecessary testing might cause a delay of the elective surgery. Nevertheless, for a DRP risk score, sensitivity (reduction of false negatives, type II error) is more important as misclassification of a high-risk patient may lead to harm [12]. Consequently, selecting a cut-off value of 0.35 for each model resulted in achieving sensitivities exceeding 77% and specificities surpassing 73% in the development and validation sets of both models.

18. **Line 312-313. Sensitivity and specificity of the 5VM achieved 77.6% (SD 0.078) respectively 76.5% (SD 0.061) with an AUC of 0.856 (SD 0.040). This statement needs to be revisited; not clear.**

- *Thank you for your comment. We revised the statement to enhance clarity:*

The 5VM demonstrated a sensitivity of 77.6% (SD 0.078) and specificity of 76.5% (SD 0.061), along with an AUC of 0.856 (SD 0.040).

19. **Discussion section needs reorganization by focusing on prediction ability of the tools identified and their implications in clinical practice.**

- *Thank you for the hint. We totally agree and reorganized this section by directly mentioning “clinical implications and future research” after the “summary” and shifting “outcomes in the context of previous research” and “strengths and limitations” to the end of the section. Furthermore, we focused on critically discussions about the prediction ability, and clinical practice implications:*

**Clinical Implications and Future Research**

In diagnostic modelling for identifying DRP in elective surgical patients, certain predictors emerged as highly influential. Notably, age and number of drugs at admission served as robust predictors in both, the 2VM and the 5VM model. The alternative 2VM model, a simplified version of the 5VM, offers practicality in situations where information, necessary for the comprehensive model (5VM) is lacking or time constraints are pressing. Predictors of the 2VM finds resonance in previous research, notably in a study, which identified medication errors in a surgical setting [13]. However, it is essential to note that the streamlined nature of the 2VM may introduce risks of underfitting, due to its limited variety of predictors potentially compromising its reliability. Thus, the choice between the 2VM and the more comprehensive 5VM is pivot, with considerations extending beyond mere convenience. While the 2VM may suffice in certain scenarios, particularly where data availability is constrained, the 5VM (additionally including BMI, gender and renal function) offers greater depth and robustness, enhancing the model’s predictive power. Hence, prudent decision-making necessitates a nuanced understanding of the trade-offs between simplicity and predictive accuracy, advocating for the utilization of the 5VM whenever feasible.

The good performances of the mediPORT versions (5VM, 2VM) with an AUC=0.856 and an AUC=0.847 respectively might help in medical and pharmaceutical decision making and assist the health care service at surgical admissions (i.e. effective planning and quality management). Both strengths might lead to improved medication and safety of care for surgical patients. Performances of mediPORT versions are on par with the performance of other DRP models. Urbina et. al. and Saldanha et al. developed a DRP prediction tool for hospitalized patients which showed a slightly lower discriminatory power of an AUC=0.776 and AUC= 0.729 respectively in the validation set [14, 15].

The effect on type I and type II errors depends on the diagnostic models and the threshold for classification (cut-off). Considering sensitivity, specificity, and threshold is crucial when evaluating predictive model performance and real-world implications. Choosing an optimal cut-off value is challenging as a model with higher sensitivity will report lower specificity and vice versa. While patients identified as false positive (type I error) may result in increased medication scrutiny, the information on risk has potential to cause unnecessary concern to patients and clinicians. Additionally, unnecessary testing might cause a delay of the elective surgery. Nevertheless, for a DRP risk score, sensitivity (reduction of false negatives, type II error) is more important as misclassification of a high-risk patient may lead to harm [12]. Consequently, selecting a cut-off value of 0.35 for each model resulted in achieving sensitivities exceeding 77% and specificities surpassing 73% in the development and validation sets of both models. Previous developed models showed reasonable sensitivity but lacked in specificity, which was lower than 62% [3, 4]. The cut-off of 0.35 for the ROC curve represents the threshold value used to classify patients into different categories based on the predicted risk of DRP. In practical terms, it means that if a patient’s predicted risk score is above 0.35, they would be classified as high risk for DRP, while those with a score below 0.35 would be classified as low risk.

In modern healthcare, it is essential to base the practical use of tools on the best available evidence. This means applying evidence on test performance with careful judgment and testing efficiently to optimize patient care. Moreover, considering the value and affordability of a test before requesting it, ensures resources are used effectively. It is also crucial to be aware of the downsides and drivers of over-diagnosis, confront uncertainties, and maintain a patient-centred approach. Addressing ethical issues and recognizing cognitive biases further enhances the quality of testing. Teaching and learning these core principles through a systematic approach ensures that healthcare professionals can navigate the complexities of testing with confidence and precision [5]. When using the mediPORT tool, clinical pharmacists can intervene during medication reconciliation and patient interviews at the PAC visit to rectify any omissions.

While predicting potential DRP provides valuable insights, it is essential to consider the clinical context and the likelihood of the predicted event to actually occur. For example, even if a patient is predicted to experience a potential DRP, such as requiring a dosage adjustment for a drug due to renal insufficiency, it may not be clinically relevant in this specific case. While diagnostic models can inform clinical decision-making, their application must be considered in conjunction with the clinical context and patient-specific factors.

Future research might evaluate the predictive performance of the algorithm in a different setting. For instance, risks of patients can be assessed retrospectively by the tool and compared to the estimated risks by pharmacists in real life practice. Results can then be used to verify sensitivity (true positive) and specificity (false positive) of the tool.

**20. The authors should describe how 2VM outperformed the 5VM given the significance of the other 3 variables in the multivariable model? This is statistically contradictory.**

- *Thank you for the important hint. Given that the model selection was based on the minimum of AIC rather than statistical significance of individual variables, it is important to accurately reflect this in the discussion. We clarified on this aspect:*

In diagnostic modelling for identifying DRP in elective surgical patients, certain predictors emerged as highly influential. Notably, age and number of drugs at admission served as robust predictors in both, the 2VM and the 5VM model. The alternative 2VM model, a simplified version of the 5VM, offers practicality in situations where information, necessary for the comprehensive model (5VM) is lacking or time constraints are pressing. Predictors of the 2VM finds resonance in previous research, notably in a study, which identified medication errors in a surgical setting [13]. However, it is essential to note that the streamlined nature of the 2VM may introduce risks of underfitting, due to its limited variety of predictors potentially compromising its reliability. Thus, the choice between the 2VM and the more comprehensive 5VM is pivot, with considerations extending beyond mere convenience. While the 2VM may suffice in certain scenarios, particularly where data availability is constrained, the 5VM (additionally including BMI, gender and renal function) offers greater depth and robustness, enhancing the model’s predictive power. Hence, prudent decision-making necessitates a nuanced understanding of the trade-offs between simplicity and predictive accuracy, advocating for the utilization of the 5VM whenever feasible.

20. **Figure 1 is not appropriately illustrated. The risk factors come before DRP.**

- *Thank you for the important hint. We totally agree upon risk factors coming before DRP. We revised the graph and visualized the timeline from the risk factors to the DRP more clearly:*

**22. Figure 3. Out of more than 11,000 patients, why did the authors select 78 participants younger than 18 years; and later excluded? What is the effect on type I and type II error?**

- *Thank you for your comment. To ensure patient inclusion of patients solely reviewed by clinical pharmacists, those under the age of 18 years were excluded. Their subsequent exclusion was imperative to maintain the study's integrity and ensure adherence to predetermined eligibility standards. We added information to enhance clarity on this aspects:*

Patients, who did not undergo review by clinical pharmacists, i.e. undergoing day care surgery and under the age of 18 years, were excluded.

The effect on type I and type II errors depends on the diagnostic models and the threshold for classification (cut-off). Considering sensitivity, specificity, and threshold is crucial when evaluating predictive model performance and real-world implications. Choosing an optimal cut-off value is challenging as a model with higher sensitivity will report lower specificity and vice versa. While patients identified as false positive (type I error) may result in increased medication scrutiny, the information on risk has potential to cause unnecessary concern to patients and clinicians. Additionally, unnecessary testing might cause a delay of the elective surgery. Nevertheless, for a DRP risk score, sensitivity (reduction of false negatives, type II error) is more important as misclassification of a high-risk patient may lead to harm [12]. Consequently, selecting a cut-off value of 0.35 for each model resulted in achieving sensitivities exceeding 77% and specificities surpassing 73% in the development and validation sets of both models. Previous developed models showed reasonable sensitivity but lacked in specificity, which was lower than 62% [3, 4]. The cut-off of 0.35 for the ROC curve represents the threshold value used to classify patients into different categories based on the predicted risk of DRP. In practical terms, it means that if a patient’s predicted risk score is above 0.35, they would be classified as high risk for DRP, while those with a score below 0.35 would be classified as low risk.

**23. Figure 5. The model developed by the authors was supposed to predict DRP; but in figure 2, they have used to predict occurrence of complications. Are all complications DRPs?**

- *Thank you for the relevant hint. We revised the figure among the probability of DRP.*

**24. Figure 7. May the authors describe what a cut-off of 0.35 mean for the ROC curve? How should the practitioners score the models in real practice? It should come out clear.**

- *Thank you for the important hint. We provided information on the ROC curve and added meanings for practical implementation:*

The cut-off of 0.35 for the ROC curve represents the threshold value used to classify patients into different categories based on the predicted risk of DRP. In practical terms, it means that if a patient’s predicted risk score is above 0.35, they would be classified as high risk for DRP, while those with a score below 0.35 would be classified as low risk.

**25. Table 1. What does adjusted p values mean? Are the ORs presented in table 1 adjusted or crude?**

- *Thank you for the comment. All P-values were adjusted for multiplicity using Bonferroni Holm (described in the Methods section). With a large number of related tests, the risk of a type II error increases (assuming there is a difference although there is not). By adjusting for this, the probability of rejecting incorrectly is kept low. However, this does not affect the odds ratios themselves. The ORs are therefore crude. Since the adjustment of the P-values is addressed in the methods section, the "adjusted" was removed from the results section to avoid confusion.*

**26. How the authors arrived at the 5 or the 2 predictors used to construct the models is not well presented in the manuscript. The multivariate regression model is not clearly presented anywhere. Though this is the backbone for model development studies.**

- *Thank you for the relevant hint. In backwards selection, different models (i.e. different compositions of predictors) were tested on the basis of their AIC, whereby the model with the lowest AIC was ultimately used. For this purpose, the model was divided into test and training data sets. The training set was used to perform the variable selection and the test set was used to test how well the model classified (calculation of sensitivity, specificity, etc.). Since the coefficients and therefore the variable selection strongly depend on the existing training set (allocation to test and training set was random), the backwards selection was carried out 1000 times, with a new allocation to training and test set each time. This was intended to ensure a robust selection of predictors, which produced the best model (according to AIC) in most combinations of training and test set. Therefore, the predictors that were preliminary in >=50% of the backwards eliminations were selected to create the final model (This was also the most common combination). In addition, it was found that 2 predictors occurred in 100% of the runs, which ultimately resulted in the 2 variable model. After the validation of the 2-variable model did not provide any bad values, it was decided to include it as a simpler alternative to the 5-variable model, as it is easier to handle in everyday clinical practice. As mentioned above, the 2 variable model runs the risk of being under fitted, which is why the 5 variable model is preferable if possible.*

*The development of the model was described mostly in the "Methods" section, which has now been expanded to make the process more transparent. The models themselves (both 5VM and 2VM) are presented in Figure 4, including their formula. This is actually truly somewhat understated, which is why the models have now been presented more prominently in the section "Development of the mediPORT risk model".*

Subsequently, for the logistic regression models predicting DRP, a backward variable selection algorithm was used, with the Akaike Information Criterion (AIC) value being used to determine the best model [16]. Because this procedure requires complete data, patients with missing data were excluded from the model development. However, if there were no missing values in the variables that were included in the final model, the data was still used for the test dataset to validate the final model. The remaining data set (without missing values) was divided by randomization into 70% training and 30% test data. Depending on the composition of the training and test set, the variables that were finally selected for inclusion in the prediction model may vary. Therefore, in order to achieve a good generalization and compensate for possible instabilities (e.g., overfitting, underfitting), the split of the data set and the subsequent selection of variables by backward elimination was repeated several times (1000x) [16]. At the same time, the EPV ratio was computed for each run to ensure that the resulting models at least respected the rule of thumb EPV >10, when estimating the parameters in the case of different compositions of the training set to avoid additional overfitting [11]. Those predictors that appeared in more than 50% of the backward eliminations were finally used to build the resulting model. These variables are also the ones that occur as combination in most of the models with the lowest AIC per run. In addition, a second model was created with those variables that occurred in 100% of the cases.

To simplify the presentation of the final mutlivariate regressions, the calculation of the individual risk was conducted in two steps. First, the risk score was calculated (either with five or two variables, depending on what is available). Next, this risk score was inserted into the exponential formula below, which finally gave the estimated risk as a percentage. This formula resulted from the conversion of the log odds of the logistic regression.

$$5VM equation for risk score:-7,29712977153007-0,394421234628523\times female\left( yes;no \right)^{*}+0,423965922272388 \times age+0,257237146449912 \times number of drugs+0,0339923294039108\times BMI+0,86186079010242 \times renal function\text{ 15-29 (yes/no) + 1,78880673311741 }\times renal function\text{ 30-59 (yes/no) + 0,862978252896248}\times renal function\text{ 60-89 (yes/no) + 1,26877761969504 }\times renal function\text{ >89 (yes/no) }$$

$$2VM equation for risk score:-5,8184055+0,423965922272388 \times age+0,257237146449912 \times number of drugs$$

$$Individual estimated risk of DRP \left( \% \right):\frac{e^{risk score}}{1+ e^{risk score}}\times100$$

*1 if “yes”, 0 if “no”

**Comments to the Author**

1. Is the manuscript technically sound, and do the data support the conclusions?

Reviewer #1: Partly

- *We revised this aspect. A language check by a natural speaker was conducted.*

2. Has the statistical analysis been performed appropriately and rigorously?

Reviewer #1: No

- *We wish to highlight the invaluable contribution of two statisticians from IDA Lab Salzburg who played an active role in the study's statistical analysis. Their efforts encompassed meticulous planning of variable selection, sample size calculation, careful consideration of model uncertainty, and thorough model validation. Furthermore, we have taken great care to address reviewer comments, particularly those pertaining to unclear wording, to ensure that the revised sections are both clear and accurate.*

3. Have the authors made all data underlying the findings in their manuscript fully available?

Reviewer #1: No

- *We have updated our Data Availability statement to better address the concerns raised by the reviewer:*

The authors are unable to publicly release the dataset due to ethical and legal restrictions (data contain potentially sensitive information). Researchers seeking access to confidential data can obtain it from the Paracelsus Medical University Salzburg by meeting the required criteria. For data requests, please contact the data protection office: [datenschutz@pmu.ac.at](mailto:datenschutz@pmu.ac.at).

4. Is the manuscript presented in an intelligible fashion and written in standard English?

Reviewer #1: No

- *We revised this aspect. A language check by a natural speaker was conducted.*

Journal requirements:

2. We noted in your submission details that a portion of your manuscript may have been presented or published elsewhere. [A manuscript addressing the nature of drug-related problems (secondary outcomes) by using a part of the same study population (case patients) was recently submitted to the European Journal of Hospital Pharmacy.] Please clarify whether this publication was peer-reviewed and formally published. If this work was previously peer-reviewed and published, in the cover letter please provide the reason that this work does not constitute dual publication and should be included in the current manuscript.

- *A manuscript addressing the prevalence and nature of drug-related problems (secondary outcomes) by using a part of the same study population (case patients) was submitted to the Journal Science Progress. We have received an acceptance from the journal. The article is now in the pre-publication process, the DOI is already available (the study is not online yet): [17]*

3. We note that you have indicated that there are restrictions to data sharing for this study. PLOS only allows data to be available upon request if there are legal or ethical restrictions on sharing data publicly. For more information on unacceptable data access restrictions, please see <http://journals.plos.org/plosone/s/data-availability#loc-unacceptable-data-access-restrictions>.

Before we proceed with your manuscript, please address the following prompts:

a) If there are ethical or legal restrictions on sharing a de-identified data set, please explain them in detail (e.g., data contain potentially identifying or sensitive patient information, data are owned by a third-party organization, etc.) and who has imposed them (e.g., a Research Ethics Committee or Institutional Review Board, etc.). Please also provide contact information for a data access committee, ethics committee, or other institutional body to which data requests may be sent.

b) If there are no restrictions, please upload the minimal anonymized data set necessary to replicate your study findings to a stable, public repository and provide us with the relevant URLs, DOIs, or accession numbers. For a list of recommended repositories, please see
<https://journals.plos.org/plosone/s/recommended-repositories>. You also have the option of uploading the data as Supporting Information files, but we would recommend depositing data directly to a data repository if possible.

We will update your Data Availability statement on your behalf to reflect the information you provide.

- *Thank you for the relevant hint. We have updated our Data Availability statement:*

The authors are unable to publicly release the dataset due to ethical and legal restrictions (data contain potentially sensitive information). Researchers seeking access to confidential data can obtain it from the Paracelsus Medical University Salzburg by meeting the required criteria. For data requests, please contact the data protection office: [datenschutz@pmu.ac.at](mailto:datenschutz@pmu.ac.at).

5. We note that Figure(s) 1, 2, 4 and 5 in your submission contain copyrighted images. All PLOS content is published under the Creative Commons Attribution License (CC BY 4.0), which means that the manuscript, images, and Supporting Information files will be freely available online, and any third party is permitted to access, download, copy, distribute, and use these materials in any way, even commercially, with proper attribution. For more information, see our copyright guidelines: <http://journals.plos.org/plosone/s/licenses-and-copyright>.
 
We require you to either (1) present written permission from the copyright holder to publish these figures specifically under the CC BY 4.0 license, or (2) remove the figures from your submission:
 
1. You may seek permission from the original copyright holder of Figure(s) 1, 2, 4 and 5 to publish the content specifically under the CC BY 4.0 license. 

- *Thank you for the important hint. We revised Figure(s) 1, 2, 4 and 5. No copyright images are included in any of the figures.*

# 31st July 2024 (rejection):

Reviewer #2:

1. **The authors propose a risk prediction tool of drug-related problems in patients submitted to elective surgery. Unfortunately, the case-control design is inappropriate for developing risk prediction tools unless the model's regression constant is corrected to provide an accurate estimate of the baseline probability of the outcome, which the authors did not do. A risk prediction tool absolutely needs an estimate of the prevalence of the outcome, and a case-control study does not provide such estimate. In the analysis done by the authors, the logistic regression model assumed that the prevalence of DRPs was 300 cases/1500 total study population = 25%, which is wrong. Therefore, all the results in this manuscript are wrong.**

- *Our study on a risk-prediction tool involves complex statistics, making it challenging to follow. We basically understand the point which has been raised by the reviewer: Indeed, in (retrospective) case-control designs, odds ratios should be used instead of, for example, relative risks, since the prevalence is in a way fixed by the design of the study. However, turning to our use case now, we are using a logistic regression model, and the coefficients of this model can be interpreted in terms of (conditional) odds ratios. Actually, it is conventional statistical wisdom that this is one of the major advantages of a logistic regression model, since it can be used for data from any study design. In addition to that, regarding risk prediction, it is definitely valid to use the logistic regression model in order to differentiate between “low risk” and “high risk” subjects, by defining a cutoff for the predicted probability. To take an example, if we say that “high risk” corresponds to a probability of having a serious adverse events > 25 percent, a person with a predicted probability of 28 percent would be classified as “high risk”, whereas a person with a predicted probability of 18 percent would be “low risk”.*

*Now, the reviewer claimed that without adjusting the baseline risk in the logistic model for the estimated prevalence, “all results are wrong”. This is definitely not true, since incorporating the estimated prevalence is not necessary at all: the probability cutoff described above implicitly accounts for that. In other words, the adjustment of the baseline model coefficient that corresponds to the prevalence only leads to a shift of the probabilities, but does not change the order at all – so, no matter what the prevalence is, our approach allows for classifying subjects into “low risk” and “high risk” patients. We would like to emphasize that the probability cutoff was chosen such that a high sensitivity is achieved, and the specificity was also shown to be good in our model validation.*

*So, for the reasons stated above, we would like to ask for another review – we are more than happy to take critical comments into account that help to improve our approach, but we just cannot agree with the statements of the previous review round, since they are not based on statistically solid grounds.*

*Based on a previous study mentioned at p. 12: “A manuscript addressing the prevalence and nature of drug-related problems (secondary outcomes) by using a part of the same study population (case patients) was submitted to the Journal Science Progress. We have received an acceptance from the journal. The article is now in the pre-publication process, the DOI is already available (the study is not online yet) [17]”, we added information on the prevalence and nature of DRP to enhance clarity:*

A prior study investigating secondary outcomes including the nature and prevalence of DRP in the target population revealed that 9.89% patients experienced at least one DRP. The primary causes of DRP were identified as drug-drug interactions (30.3%) and supra-therapeutic dosing (18.0%) [17].

**References:**

1.	Roten I, Marty S, Beney J. Electronic screening of medical records to detect inpatients at risk of drug-related problems. Pharmacy World & Science. 2010;32(1):103-7. doi: 10.1007/s11096-009-9352-6.

2.	Saedder EA, Lisby M, Nielsen LP, Rungby J, Andersen LV, Bonnerup DK, et al. Detection of Patients at High Risk of Medication Errors: Development and Validation of an Algorithm. Basic Clin Pharmacol Toxicol. 2016;118(2):143-9. Epub 2015/08/25. doi: 10.1111/bcpt.12473. PubMed PMID: 26299815.

3.	Tangiisuran B, Scutt G, Stevenson J, Wright J, Onder G, Petrovic M, et al. Development and validation of a risk model for predicting adverse drug reactions in older people during hospital stay: Brighton Adverse Drug Reactions Risk (BADRI) model. PloS one. 2014;9(10):e111254. Epub 2014/10/31. doi: 10.1371/journal.pone.0111254. PubMed PMID: 25356898; PubMed Central PMCID: PMCPMC4214735.

4.	Geeson C, Wei L, Franklin BD. Development and performance evaluation of the Medicines Optimisation Assessment Tool (MOAT): a prognostic model to target hospital pharmacists' input to prevent medication-related problems. BMJ quality & safety. 2019;28(8):645-56. Epub 2019/03/09. doi: 10.1136/bmjqs-2018-008335. PubMed PMID: 30846489; PubMed Central PMCID: PMCPMC6716361.

5.	Power M, Fell G, Wright M. Principles for high-quality, high-value testing. Evidence-based medicine. 2013;18(1):5-10. Epub 2012/06/29. doi: 10.1136/eb-2012-100645. PubMed PMID: 22740357; PubMed Central PMCID: PMCPMC3585491.

6.	Kaufmann CP, Stampfli D, Hersberger KE, Lampert ML. Determination of risk factors for drug-related problems: a multidisciplinary triangulation process. BMJ Open. 2015;5(3):e006376. Epub 2015/03/22. doi: 10.1136/bmjopen-2014-006376. PubMed PMID: 25795686; PubMed Central PMCID: PMCPMC4368979.

7.	Botelho SF, Neiva Pantuzza LL, Marinho CP, Moreira Reis AM. Prognostic prediction models and clinical tools based on consensus to support patient prioritization for clinical pharmacy services in hospitals: A scoping review. Research in Social and Administrative Pharmacy. 2021;17(4):653-63. doi: <https://doi.org/10.1016/j.sapharm.2020.08.002>.

8.	PCNE. Classification for Drug related problems V 9.1 2020 [05.06.2023]. Available from: <https://www.pcne.org/upload/files/417_PCNE_classification_V9-1_final.pdf>.

9.	Woodward M. Epidemiology: Study Design and Data Analysis, Third Edition: CRC Press; 2013.

10.	Iwagami M, Shinozaki T. Introduction to Matching in Case-Control and Cohort Studies. Annals of Clinical Epidemiology. 2022;4(2):33-40. doi: 10.37737/ace.22005.

11.	Moons KG AD, Reitsma JB, Ioannidis JP, Macaskill P, Steyerberg EW, Vickers AJ, Ransohoff DF, Collins GS. Transparent Reporting of a multivariable prediction model for Individual Prognosis Or Diagnosis (TRIPOD): Explanation and Elaboration. . Ann Intern Med 2015;162(1):W1-W73.

12.	Falconer N, Barras M, Cottrell N. Systematic review of predictive risk models for adverse drug events in hospitalized patients. Br J Clin Pharmacol. 2018;84(5):846-64. Epub 2018/01/18. doi: 10.1111/bcp.13514. PubMed PMID: 29337387; PubMed Central PMCID: PMCPMC5903258.

13.	Nguyen TL, Leguelinel-Blache G, Kinowski JM, Roux-Marson C, Rougier M, Spence J, et al. Improving medication safety: Development and impact of a multivariate model-based strategy to target high-risk patients. PloS one. 2017;12(2):e0171995. Epub 2017/02/14. doi: 10.1371/journal.pone.0171995. PubMed PMID: 28192533; PubMed Central PMCID: PMCPMC5305217.

14.	Urbina O, Ferrandez O, Grau S, Luque S, Mojal S, Marin-Casino M, et al. Design of a score to identify hospitalized patients at risk of drug-related problems. Pharmacoepidemiol Drug Saf. 2014;23(9):923-32. Epub 2014/05/13. doi: 10.1002/pds.3634. PubMed PMID: 24817497.

15.	Saldanha V, Araujo IB, Lima S, Martins RR, Oliveira AG. Risk factors for drug-related problems in a general hospital: A large prospective cohort. PloS one. 2020;15(5):e0230215. Epub 2020/05/06. doi: 10.1371/journal.pone.0230215. PubMed PMID: 32369489; PubMed Central PMCID: PMCPMC7199929.

16.	Kuhn M. Building Predictive Models in R Using the caret Package. Journal of Statistical Software. 2008;28(5):1 - 26. doi: 10.18637/jss.v028.i05.

17.	Simon C, Rose O, Kanduth K, Pachmayr J, Clemens S. Drug-related problems in elective surgical inpatients: A retrospective study. Science Progress. 2024;107(3) 1–15. doi: 10.1177/00368504241263534.
